# Single-Labeled Oligonucleotides Showing Fluorescence Changes upon Hybridization with Target Nucleic Acids

**DOI:** 10.3390/molecules23010124

**Published:** 2018-01-08

**Authors:** Gil Tae Hwang

**Affiliations:** Department of Chemistry and Green-Nano Materials Research Center, Kyungpook National University, Daegu 41566, Korea; giltae@knu.ac.kr; Tel.: +82-53-950-5331; Fax: +82-53-950-6330

**Keywords:** DNA, nucleic acids chemistry, oligonucleotides, fluorescent probes, single-nucleotide polymorphism, fluorophore

## Abstract

Sequence-specific detection of nucleic acids has been intensively studied in the field of molecular diagnostics. In particular, the detection and analysis of single-nucleotide polymorphisms (SNPs) is crucial for the identification of disease-causing genes and diagnosis of diseases. Sequence-specific hybridization probes, such as molecular beacons bearing the fluorophore and quencher at both ends of the stem, have been developed to enable DNA mutation detection. Interestingly, DNA mutations can be detected using fluorescently labeled oligonucleotide probes with only one fluorophore. This review summarizes recent research on single-labeled oligonucleotide probes that exhibit fluorescence changes after encountering target nucleic acids, such as guanine-quenching probes, cyanine-containing probes, probes containing a fluorophore-labeled base, and microenvironment-sensitive probes.

## 1. Introduction

Since the post-genome era, there has been a growing need for faster and more accurate detection methods for nucleic acids. In particular, the interest in single-nucleotide polymorphisms (SNPs), which involve a variation of a single nucleotide at a specific location in the genome, is increasing, so typing an SNP using a DNA probe is very important [1,2,3,4]. Several methods for SNP typing have been developed in efforts to establish an ideal typing system that exhibits very sensitive and specific behavior for the target DNA, without costly and time-consuming steps. Among these methods, molecular beacon (MB) probes have been widely used for the detection of SNPs, for the real-time detection of nucleic acids, for the quantification of polymerase chain reactions (PCRs), for isothermal amplification, as DNA microarray–immobilized probes, and as antisense probes for detecting RNA in vivo [5,6,7,8].

**Figure 1:**
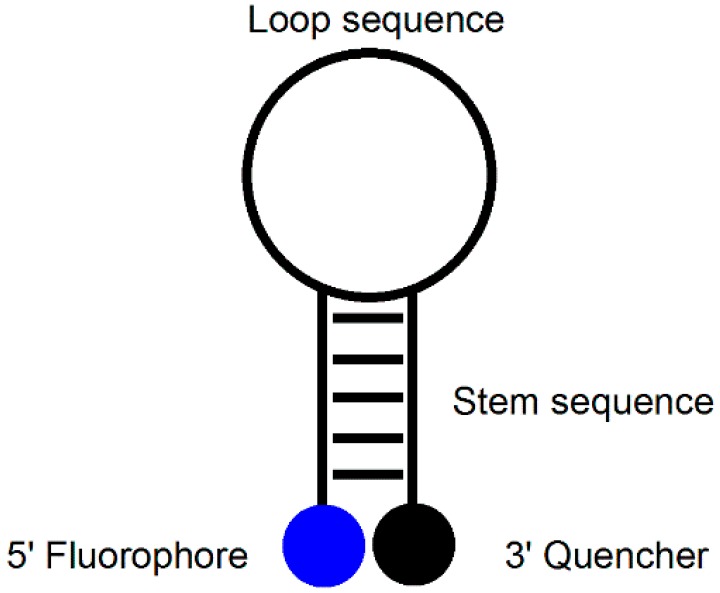
MB probe.

An MB probe is an oligonucleotide that forms a hairpin-like stem-loop structure tagged with a fluorophore at its 5′-terminus and a quencher at its 3′-terminus (Figure 1). The hairpin loop consists of approximately 15–25 nucleotides (nt) that are complementary to the target DNA, and the terminal stems are composed of 5–7 nt that are complementary to each other [9,10,11]. When the MB probe exists in the form of a hairpin structure, the fluorescence disappears because the quencher is positioned close to the fluorophore. When the MB probe meets its target DNA, however, the hairpin structure is opened, and fluorescence is restored as a result of the separation of the fluorophore and quencher units. The advantage of MB probes over single-dye probes (linear probes) is that they provide high signal-to-noise ratios and high degrees of single-base mismatch discrimination. Despite these attractive features, MB probes have a disadvantage in that they require a specific target sequence of 15–25 nt to separate the fluorophore and quencher upon dimerization with the target. In addition, because the hairpin must be opened, the rate of dimer formation is slower than that of a corresponding linear probe. In addition, both the fluorophore and quencher units are required, adding to the cost of preparation.

If fluorescent hairpin- and linear-oligonucleotides could be made to function in a manner similar to that of MB probes, but without the need for quencher units, such probes would be simpler and cheaper to prepare. Furthermore, quencher-free DNA probes could then be prepared with the fluorophore positioned anywhere along the sequence, not only at the end of the stem. Examples of systems capable of fluorescence-based sequence detection, discrimination of alleles, and DNA quantification using oligonucleotide probes containing only fluorophore units (i.e., without quenchers) have been reported. This article reviews recent progress in the development of single-labeled DNA probes that can sense the presence of a specific nucleic acid through a change in fluorescence intensity.

## 2. Guanine-Quenching Probes

**Figure 2:**
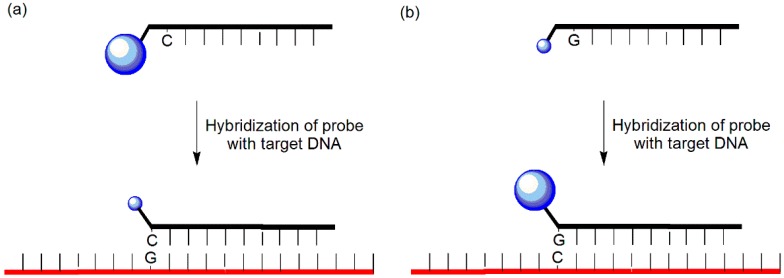
(**a**) Guanine-quenching probe and (**b**) Guanine-dequenching probe.

Fluorescence quenching between a guanosine residue and a fluorophore is commonly exploited because guanine bases function as energy acceptors [12,13,14]. Two types of probes have been designed using this principle (Figure 2). First, quenching of the fluorophores such as fluorescein, BODIPY, 6-carboxyfluorescein, and tetramethyl-6-carboxyrhodamine can be achieved by placing one or more adjacent cytosine residues next to the fluorophore unit of the probe [15,16,17]; hybridization induces a decrease in the fluorescence of the fluorophore close to one or more guanine residues in the complementary strand. Second, dequenching can be induced by placing one or more guanine residues next to the fluorescein unit in the probe [18]; the internal quenching is relaxed upon hybridization with the target DNA, thereby enhancing the emission.

## 3. Cyanine-Containing Probes

As mentioned above, the interaction of fluorophores with nucleobases usually leads to the quenching of fluorescence. However, some cyanines such as thiazole orange (**TO**) and oxazole yellow (**YO**) are notable exceptions because they are powerful reagents for nucleic acid staining. In attempts to develop fluorescence “turn-on” probes, oligonucleotides containing these dyes have been developed [19,20,21,22,23,24,25].

**Figure 3:**
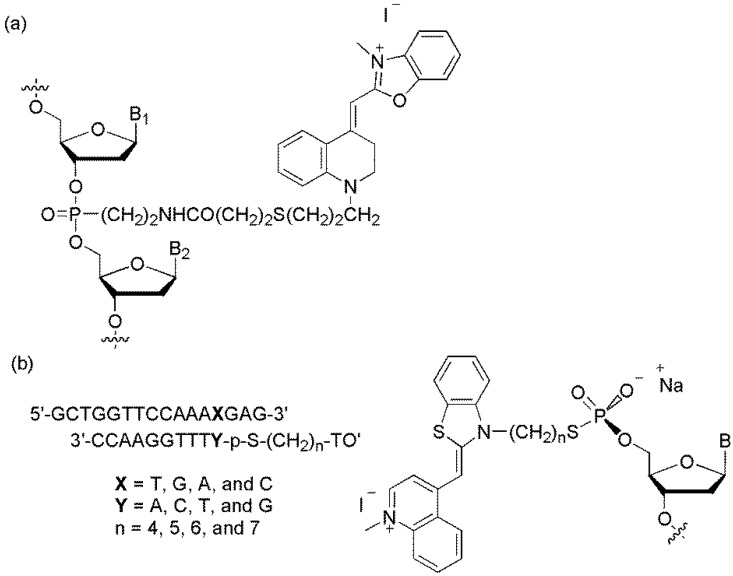
(**a**) **YO**-linked probe and (**b**) **TO**-linked probe.

The Ishiguro group designed a **YO**-linked DNA probe [19]. A **YO** moiety was inserted at an internal cytidine, C*, of a 13mer, 5′-CTCGC*GGGGGCTG-3′, complementary to the 5′-terminus non-coded region of hepatitis C virus RNA (Figure 3a). The **YO**-linked DNA probe recognized the complementary DNA or RNA and increased the fluorescence by the intercalation of the **YO** moiety into the target DNA or RNA.

The Asseline group reported a **TO**-linked DNA probe (Figure 3b) [20]. The fluorescence intensities of the mismatched duplexes were greater than those of the corresponding matched duplexes. The highest discrimination factor, which was the fluorescence intensity ratio between the mismatched duplexes and the perfectly matched ones, was obtained when **TO** was linked in the position adjacent to A (discrimination factor >4) and to T (discrimination factor >3).

**Figure 4:**
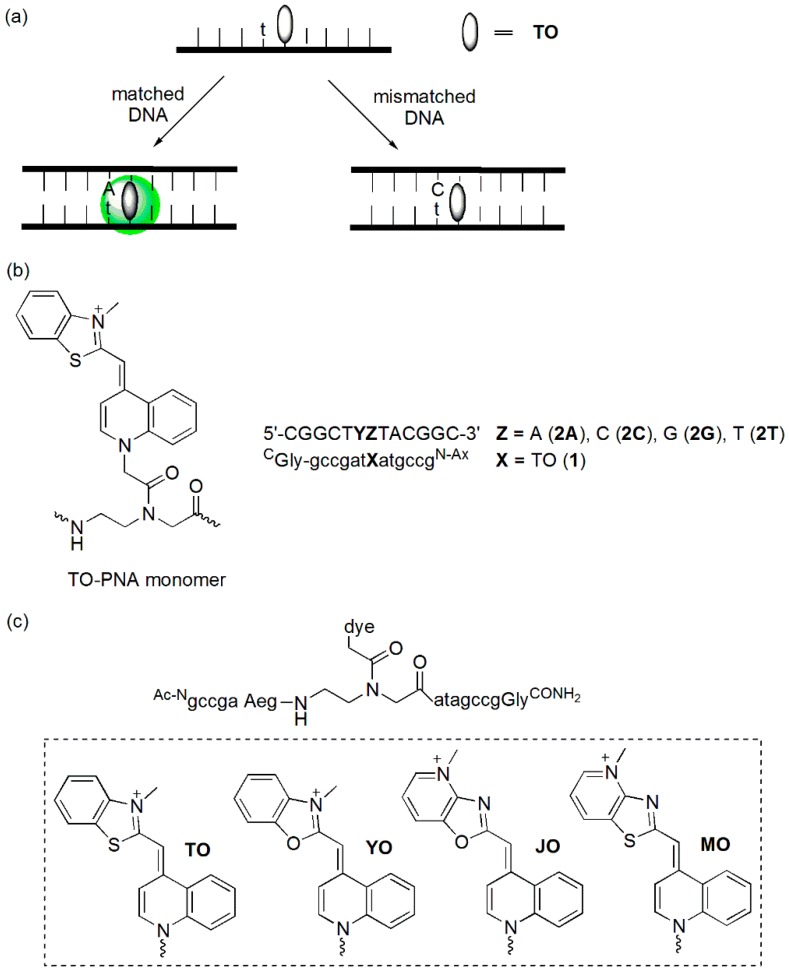
(**a**) Principle of FIT-probes; (**b**) **TO**-PNA conjugate; and (**c**) **TO**-, **YO**-, **MO**-, and **JO**-PNA conjugates.

The Seitz group developed forced intercalation probes (FIT probes, Figure 4a), **TO**-linked PNA (peptide nucleic acid) probes, in which **TO** was utilized as a fluorescent base surrogate [21,22,23]. When these probes were hybridized to the matched DNA target, hybridization-induced fluorescence enhancement was observed. **TO** fluorescence was sensitive to the structural disturbance of PNA/DNA caused by an adjacent base mismatch (Figure 4b). The emission of a duplex (**1**/**2C**, Y = C) containing a C/T mismatch was reduced 3.4- and 11-fold relative to that of a matched duplex (**1**/**2A**, Y = A) when measured at 25 °C and 61 °C, respectively. The discrimination factor at 61 °C was higher because the thermal stability of the mismatch duplex was 9 K lower compared to that of the matched duplex.

Other cyanine dyes such as oxazole yellow (**YO**), thiazolopyridine (**MO**), and oxazolopyridine (**JO**) have also been tested (Figure 4c) [24]. The **TO**-PNA probe was able to discriminate the a/T matches from a/A and a/G mismatches with 3-fold selectivity. However, it was not able to discriminate a/C mismatches. This type of mismatch could be easily discriminated by the **YO**–PNA probe. However, the **YO**-PNA probe was not able to discriminate an a/A mismatch. Discrimination of the match/mismatch at 60 °C increased to 4- to 6-fold for the **TO**–PNA probe and to 7-fold for the **YO**-PNA probe. A glycerol-**TO** nucleotide-containing DNA probe has also been developed [25]. Emission of the matched duplex was at least 5.6-fold higher than those of mismatched duplexes at 25 °C. It was proposed that the increase in the available space introduced by mismatched base-pairs reduced the emission of the “**TO**-nucleobase”.

## 4. Probes Containing a Fluorescent Nucleobase Analog

The classical fluorescent nucleobase consists of an adenine analog 2-aminopurine that maintains complementarity to thymine but also wobble pairs with cytosine. It has a high fluorescent quantum yield but shows reduced fluorescence following its interaction with the base when inserted into the oligonucleotide [26]. Thus, fluorescent base analogs that are structurally similar to native nucleobases, capable of pairing with Watson-Crick pairs, and applicable as SNP probes are being developed.

**Figure 5:**
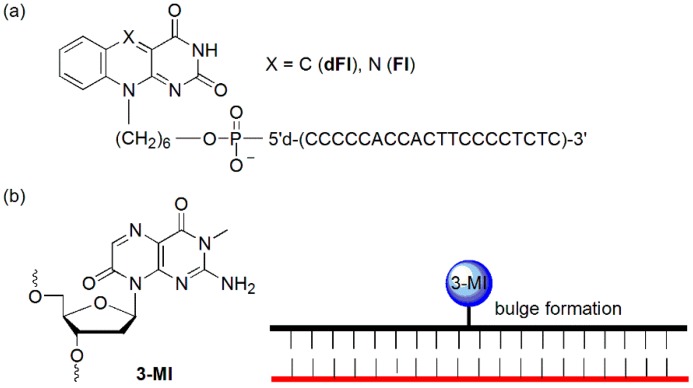
(**a**) Oligonucleotide conjugates of 5-deazaflavin (**dFl**) and flavin (**Fl**) and (**b**) single-base bulge formation of a **3-MI**-containing oligonucleotide.

The Fontecave group demonstrated that flavin (**Fl**) and deazaflavin (**dFl**) were able to hybridize to a matched target DNA both in the solution and on a solid surface and that the hybridization could be detected because of the dramatic quenching of the matched target DNA (Figure 5a) [27]. Significantly decreased quenching of fluorescence could be detected when mismatched targets were hybridized. This effect probably resulted from a combination of the increased distance between the dye and target and reduced amounts of the hybridized probe at equilibrium, making it possible to detect mutations in solution and on a solid support.

The Hawkins group reported **3-MI**-containing hybridization probe (Figure 5b) [28]. Following the formation of a matched duplex, a bulge formed at the sites of **3-MI**, leading to an increase of up to 27-fold in fluorescence intensity. They demonstrated that the bulge-formation technique could be used to detect positive PCR products using an HIV-1 detection system.

**Figure 6:**
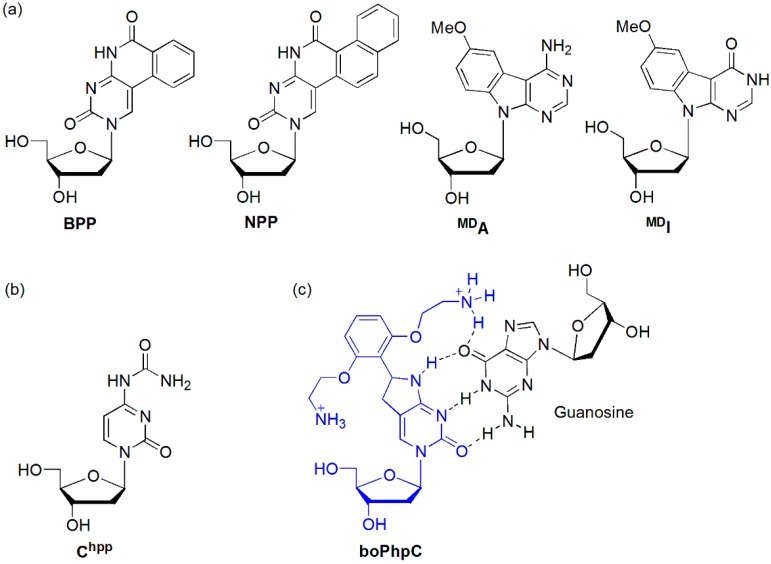
(**a**) BPP, NPP, ^MD^A, and ^MDI^, (**b**) C^hpp^, and (**c**) boPhpC.

The Saito group designed benzopyridopyrimidine (**BPP**) as an effective degenerate base, forming stable base pairs in the Watson-Crick pairing mode for **BPP**/G and in the wobble mode for **BPP**/A (Figure 6a) [29]. However, the fluorescence behavior of the **BPP**-containing oligodeoxynucleotide (ODN) [ODN(**BPP**)] was strongly dependent on the purine bases opposite **BPP**. The fluorescence quantum yield (*Φ*_F_) of ODN(**BPP**)/ODN(G) (i.e., when BPP met G, *Φ*_F_ = 0.0018) was approximately 20-fold less than that observed for ODN(**BPP**)/ODN(A) (*Φ*_F_ = 0.035). Therefore, **BPP**-containing ODN is an effective probe for typing the A/G SNP. **BPP**-containing ODN was also applied for the detection of a single nucleotide alteration in RNA [30]. ODN(**BPP**)/RNA(A) exhibited a pale blue fluorescence that was clearly distinguished from the very weak emission observed for ODN(**BPP**)/RNA(G). Naphthopyridopyrimidine (**NPP**) also could clearly distinguish the nucleobases opposite **NPP**, particularly between the A and G bases [31]. The fluorescence quantum yield of ODN(**NPP**)/ODN(G) (*Φ*_F_ = 0.007) was approximately 14-fold less than that observed for ODN(**NPP**)/ODN(A) (*Φ*_F_ = 0.096). For the other mismatched duplexes ODN(**NPP**)/ODN(C) and ODN(**NPP**)/ODN(T), the emissions were weak compared with that for ODN(**NPP**)/ODN(A) (*Φ*_F_ = 0.021 and 0.051, respectively). In addition, methoxybenzodeazaadenine (**^MD^A**) and methoxybenzodeazainosine (**^MD^I**) emitted strong fluorescence only when the base on the complementary strand was C and T, respectively [32].

The Sekine group synthesized a bicyclic 4-*N*-carbamoyldeoxycytidine derivative (**C^hpp^**) as the geometrically locked nucleoside and inserted it at the central position of a 13mer ODN (5′-CGCAAT **C^hpp^**TAACGC-3′) (Figure 6b) [33]. They found that **C^hpp^** forms stable base pairs with not only the complementary guanine base but also the adenine base. Although the emission intensity was very similar to that of the single-stranded ODN (ssODN) containing **C^hpp^** when the **C^hpp^** base faced a mismatch base A, the emission of the duplex containing a **C^hpp^**/G base pair was significantly reduced.

The Hudson group synthesized [bis-*ortho*-(aminoethoxy)phenyl]pyrrolocytosine (**boPhpC**), capable of additional hydrogen bonding with guanine, and a **boPhpC**-containing PNA (GTAGAT C**X**CT-Lys, **X** = **boPhpC**) (Figure 6c) [34]. This PNA exhibited a significant increase in affinity toward matched DNA and RNA (Δ*T*_m_ = +11.5 and +10.0 relative those of natural PNA/DNA and PNA/RNA duplexes, respectively) presumably due to an additional hydrogen bond to guanine. Interestingly, duplex formation with mismatch DNAs showed a sharp decrease in *T*_m_ (Δ*T*_m_ ≥ −13.5 °C). The discrimination was equal to or greater than that of cytosine itself. However, upon duplex formation, the quantum yield of the matched PNA/DNA duplex dramatically decreased by ca. 50% compared to that observed for a single-stranded PNA.

The Tor group designed a fluorescent nucleoside, 7-aminoquinazoline-2,4-(1*H*,3*H*)-dione, which contains an electron-rich ring fused into an electron-deficient pyrimidine, and incorporated it into the central position (**X**) of ODN (5′-GCGATG**X**GTAGCG-3′) [35]. When this nucleoside formed duplexes with A, T, and C, a quenched emission was observed, but when it met with G, a fluorescence increase of approximately 2-fold was observed. Therefore, this nucleoside sensed mismatched pairings by displaying a G-specific fluorescence enhancement, a characteristic not observed in other fluorescent nucleosides.

**Figure 7:**
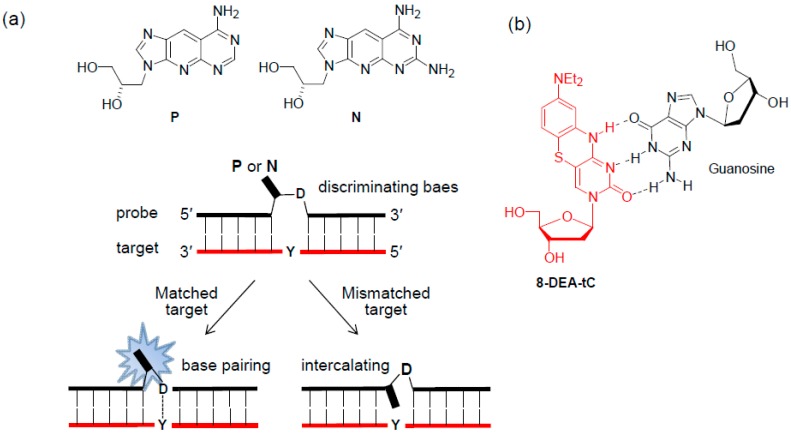
(**a**) Tricyclic base-linked acyclonucleoside, **P** and **N**, and (**b**) tricyclic cytidine analog **8-DEA-tC**.

The Ueno group reported a fluorescent tricyclic base-linked acyclonucleoside, **P** and **N**. (Figure 7a) [36,37]. When a discriminating base **D** of the probe was complementary to the DNA target base **Y**, the base pairs between **D** and **Y** caused the fluorescent analog to flip outside of the DNA helix, enhancing the fluorescence intensity of the analogs. However, when the target base **Y** was mismatched with the discriminating base **D**, the analogs intercalated into the DNA helix resulted in a decrease in the emission of the analogs. The results of the fluorescence experiment for the RNA targets were similar to those observed for the DNA targets.

The Purse group designed the tricyclic cytidine analog **8-DEA-tC** and incorporated it into the **X** of ODN, 5′-CGCAN**X**N′TCG-3′ (N and N′ = A, T, G, or C) (Figure 7b) [38]. The ssODNs were up to 5-fold brighter than the **8-DEA-tC** nucleoside and showed further emission increases in all sequences of up to 4-fold when forming a matched duplex with the complementary DNA target. However, mispairing **8-DEA-tC** with A resulted in an emission increase of less than 2-fold.

**Figure 8:**
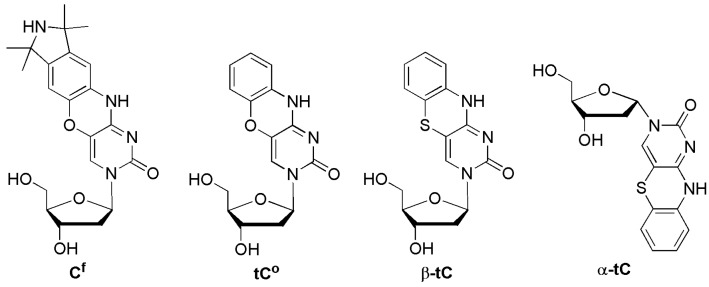
Tricyclic cytidine analogs, **C^f^**, **tC^o^**, **β-tc**, and **α-tc**.

The Sigurdsson group designed the ssODN 5′-GACCTCG**C^f^**ATCGTG-3′ containing another cytidine analog **C^f^**. Mispairing **C^f^** with A resulted in the most fluorescent among all duplexes and was similar to that of the single strand (Figure 8) [39]. The fluorescence emission of the mismatched duplexes **C^f^**/C and **C^f^**/T was between that of **C^f^**/A and the matched duplex **C^f^**/G. They also investigated the SNP typing of α- and β-anomers of phenotiazine (**tC**), which is a highly fluorescent DNA base analog that forms Watson-Crick base pairs with guanine [40] and acts as an i-motif probe [41]. Neither anomer of **tC** was suitable for SNP detection because they were unable to detect mismatches in any tested sequence [42]. However, phenoxazine (**tC^o^**) was able to detect mismatches for some flanking sequences, but **tC^o^** did not perform as good as the **C^f^** probe.

## 5. Probes Containing a Fluorophore-Labeled Base

### 5.1. HyBeacon Probes

**Figure 9:**
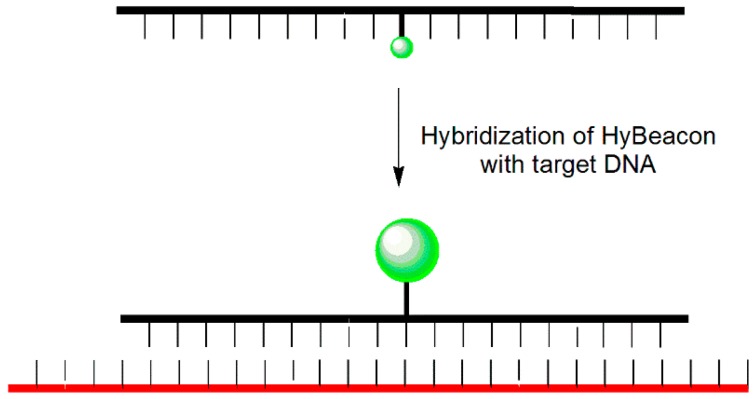
HyBeacon probe.

HyBeacon probes are linear oligonucleotides containing a fluorophore-labeled uracil base at an internal position of the oligonucleotide (no quencher moiety) and a 3′-phosphate or octanediol to prevent PCR extension (Figure 9) [43]. The fluorescent dyes 6-carboxyfluorescein, tetrachloro-6-carboxyfluorescein, and hexachloro-6-carboxyfluorescein can be attached to the 5-position uracil base through novel linkage chemistries. Hybridization of HyBeacons with complementary target DNA increases the fluorescence intensity. HyBeacon probes can be integrated into real-time PCR analysis to detect the presence and monitor the accumulation of specific DNA targets. In addition, sequence differences as small as a single nucleotide can be detected and differentiated by measuring the melting temperature.

### 5.2. Probes Containing a Nucleobase-Labeled Fluorophore with an Acetylene Group

**Figure 10:**
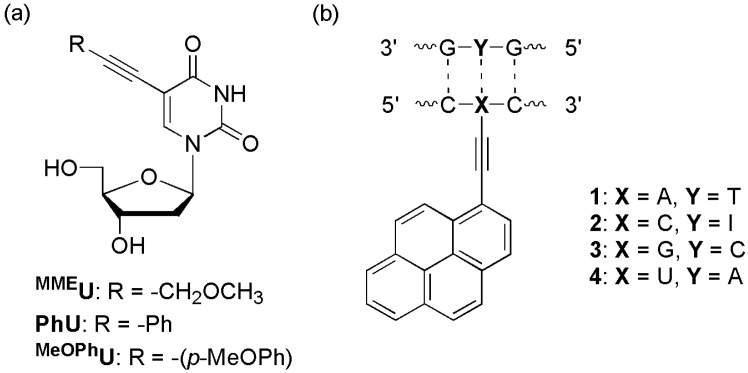
(**a**) 5-Phenylethynyluridine and (**b**) pyrenylethynyl derivatives.

The Hudson group synthesized structurally simple 5-phenylethynyl derivatives of uracil, **^MME^U**, **^Ph^U**, and **^MeO^PhU**, which are intrinsically fluorescent, and incorporated them into the **X** (5′-CGCAAT**X**TAACGC-3′) (Figure 10a) [44]. These ODNs exhibited 6-, 2-, and 6-fold increases in fluorescence intensity in the presence of complementary DNA, respectively. Although there was no significant reduction of fluorescence in the presence of mismatched DNAs, these fluorescent nucleosides were responsive to their local structure/environment and therefore have potential use in SNP probes.

The Wagenknecht group designed 1-ethynylpyrene-labeled pyrimidines and purines (Figure 10b) [45]. A strong new absorption band appeared at ~420 nm (**1** and **3**) or ~400 nm (**2** and **4**) following DNA-duplex formation. This absorption band may originate from the ground-state interaction of the 1-ethynylpyrene moiety with the adjacent base pairs (C/G). When a complementary DNA hybridized, the fluorescence intensity increased up to 40-fold relative to ssODN (in case of **3**). This difference in fluorescence intensity was highest when the duplex was excited at ~420 nm (**1** and **3**) and ~400 nm (**2** and **4**).

**Figure 11:**
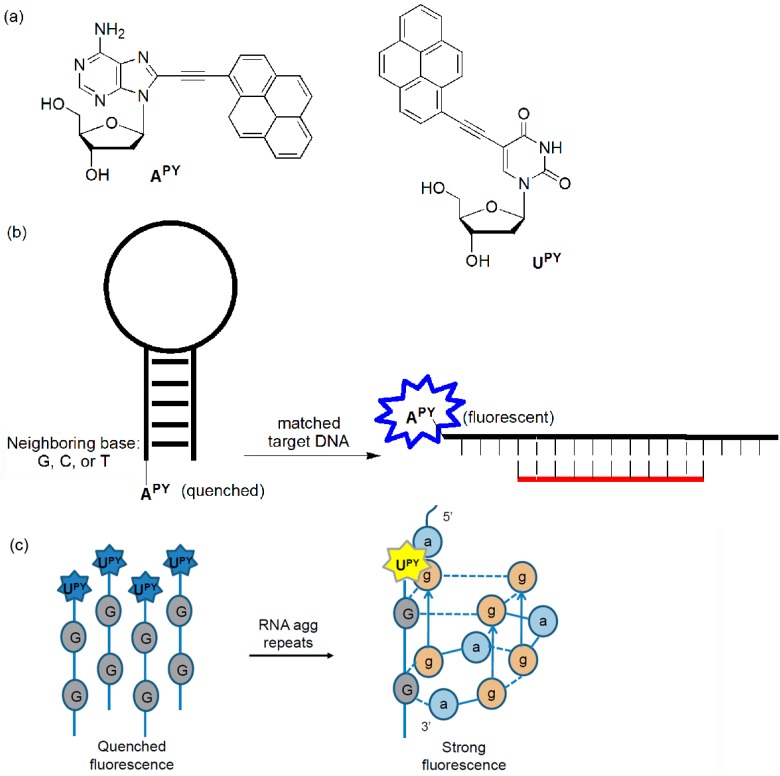
(**a**) **A^PY^** and **U^PY^**, (**b**) **A^Py^**-labeled probe, and (**c**) probe for agg trinucleotide repeats.

The Kim group incorporated pyrene-labeled deoxyadenosine (**A^PY^**) into the 5′-end of hairpins and examined the quenching effect of the neighboring bases (Figure 11). The fluorescence could be quenched through photoinduced electron transfer (PET) from the fluorophore to neighboring C, T, and G bases, but not to the A moiety. Their quenched emissions were recovered when they met the matched targets [46]. They also developed a sensitive system for detecting AGG trinucleotide repeats through the formation of intermolecular G-quadruplexes in the presence of added K^+^ ions using a ssODN (5′-**U^PY^**GGTT-3′) (Figure 11c) [47]. When this probe interacted with the RNA target sequence (5′-aggaggagga-3′), very strong fluorescence enhancements were observed (44.7-fold increase in fluorescence). In the presence of the DNA target sequence (5′-AGGAGGAGGA-3′), a large increase in fluorescence was also observed (35.0-fold).

The Hrdlicka group found that introduction of LNA nucleotides as direct neighbors into **U^PY^** improved the discrimination of SNPs [48]. They synthesized a ssODN, 5′-GTGN**U^Py^**N TGC-3′ (N = DNA nucleotide, A or LNA nucleotide, a). Hybridization of LNA-free ssODN with complementary DNA and RNA resulted in approximately 1.3- and 2.7-fold increases in emission relative to ssODN at 460 nm, respectively. Higher relative increases were observed when LNA nucleotides were incorporated as direct neighbors (approximately 2.0- and 4.5-fold increases for a duplex with complementary DNA and RNA, respectively). In contrast, mismatched duplexes were consistently less emissive than matched duplexes when using ssODN bearing the flanking LNA nucleotides. This strongly suggests that flanking LNA nucleotides can be used to produce probes with greater diagnostic potential. However, **A^PY^** was also investigated in the same way, but its ability as a SNP probe was very limited.

**Figure 12:**
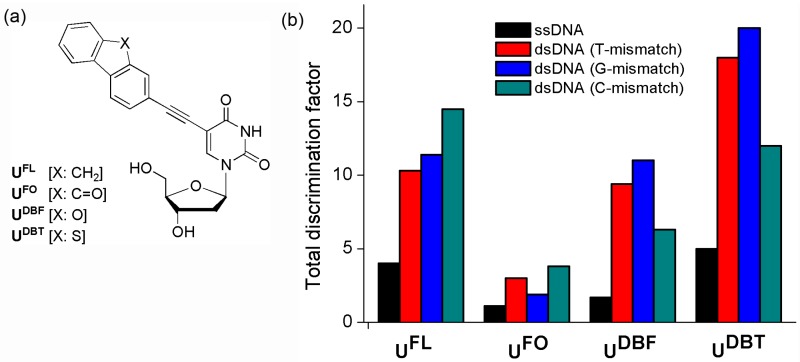
(**a**) 2′-Deoxyuridines labeled with **FL** derivatives and (**b**) Total discrimination factors for the OND4(**U^F^**) probes relative to those of ssDNAs and mismatched dsDNAs.

We have developed quencher-free MB probes that incorporate a 2-ethynylfluorene derivative covalently attached to a 2′-deoxyuridine residue (**U^F^**) (Figure 12). Although fluorene (**FL**) derivatives provide a high fluorescence yield, they do not significantly affect the stability of the DNA duplexes because they have small volumes [49,50,51,52,53,54,55]. When an **FL** derivative was introduced at the C-5 position of deoxyuridine, the DNA containing it had little effect on the stability of the double-stranded DNA (dsDNA) formed upon hybridization with the complementary DNA [56,57,58,59]. In addition, an acetylene bridge provided strong electronic bonding between the uridine moiety and the **FL** derivative.

Deoxyuridine derivatives were labeled with **FL** units [60,61,62] as well as several **FL** derivatives: 2-ethynyl-9*H*-fluoren-9-one (**FO**) [63], dibenzofuran (**DBF**) [64], and dibenzothiophene (**DBT**) [64]. **FL**, **FO**, **DBF**, and **DBT** have dramatically different photophysical properties because different atoms connect the two aromatic rings. To examine the effect of the flanking bases (FBs) on the emission properties, we modified such ODNs mainly at the bases flanking the **U^F^** units. These ODNs were 15mers containing a **U^F^** residue at the central position (Table 1).

In particular, ODN4(**U^FL^**) bearing C-FBs produced more efficient fluorescence ON/OFF systems than did other ODNs having other combinations of FBs [62]. The intensities of the fluorescence of the matched dsDNA formed between ODN4(**U^FL^**) and ODN4′(A) were increased 4.0-, 10.3-, 11.4-, and 14.5-fold over those of ODN4(**U^FL^**) and the mismatched dsDNAs with T-, G-, and C-mismatched targets, respectively. However, hybridization of the ODN4(**U^FO^**) bearing C-FBs with the matched target ODN4′(A) resulted in only a 1.1-fold increase in emission intensity relative to that of ssODN4(**U^FO^**); its total discrimination factors for the recognition of A/T, A/G, and A/C single-base mismatches were 3.0, 1.9, and 3.8, respectively [63]. The **DBF**- and **DBT**-labeled deoxyuridines **U^DBF^** and **U^DBT^**, respectively, were also introduced at the central positions of ODNs [64]. “Turn-on” responses to the matched targets were observed when the **U^DBF^** and **U^DBT^** units of ODNs bearing pyrimidine-FBs were positioned opposite to the four natural nucleobases. The total discrimination factors of the ODN2(**U^DBF^**) probe bearing T-FBs for recognition of A/T, A/G, and A/C single-base mismatches were 3.2, 7.0, and 2.8, respectively; those of the ODN2(**U^DBT^**) probe were 2.6, 4.8, and 2.3, respectively. Interestingly, the fluorescence emissions of ODN4(**U^DBF^**) and ODN4(**U^DBT^**) bearing C-FBs decreased greatly upon hybridization with all the mismatched targets, leading to total discrimination factors of 9.4 (T), 6.3 (C), and 11 (G) for the ODN4(**U^DBF^**) probe and 18 (T), 12 (C), and 20 (G) for the ODN4(**U^DBT^**) probe. In comparison, the fluorescence intensity ratios of matched dsDNAs bearing C-FBs to ssDNAs were 4.0 for **FL**, 1.1 for **FO**, 1.7 for **DBF**, and 5.0 for **DBT** (Figure 12b). Therefore, ODN4(**U^DBT^**) bearing C-FBs represent a very efficient fluorescent “turn-on” system and are more sensitive than any other quencher-free MB system.

**Figure 13:**
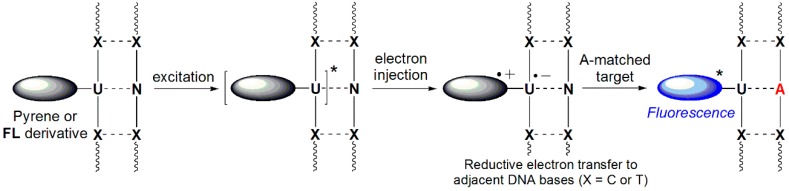
Working mechanism of quencher-free linear beacon systems.

Based on data from established studies on the reducibility of nucleobases, excitation of ODNs containing 2′-deoxyuridine units labeled with pyrene or fluorene derivatives results in electron injection into DNA, producing pyrenyl- or fluorenyl-radical cations and uracyl radical anions. Photoexcited pyrene and **FL** derivatives are capable of reducing only their adjacent pyrimidine bases (C or T), resulting in substantial quenching of emissions through base-to-base electron transfer [65,66,67,68,69,70]. Therefore, efficient quenching of ssDNAs containing a pyrene or **FL** derivative can be obtained by placing C or T nucleobases adjacent to the pyrene or **FL** derivative. This quenching is, however, inhibited when encountering a matched target, resulting in the intrinsic emission intensity of the pyrene or **FL** derivative (Figure 13). In addition, ODN4(**U^F^**) bearing C-FBs exhibited significantly decreased fluorescence emission when hybridized with all mismatched targets relative to that of the ssODN4(**U^F^**). This dramatic quenching may have resulted from the close proximity of the **FL** derivative to the two guanine units that were the complementary bases of the C-FBs and served as internal quenchers [12,13,14]. That is, ODN4(**U^F^**) bearing C-FBs could effectively discriminate mismatched targets by decreasing the fluorescence intensity as a result of the guanine bases acting as internal quenchers as well as photoinduced charge transfer to the C-FBs.

### 5.3. BDF Probes

The Saito group devised a novel strategy using pyrene-labeled base-discriminating fluorescent (BDF) oligonucleotides as probes for the discrimination of single-base alterations. The concept is based on the fluorescence change of the BDF base, which can be used to clearly distinguish the type of base in the opposite strand.

**Figure 14:**
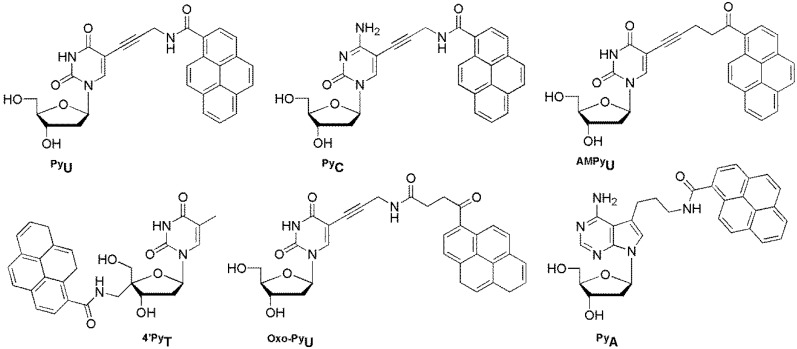
BDF probes.

**^Py^U** and **^Py^C** showed unique fluorescence properties depending on the nature of the base of the complementary strand and showed a large increase in fluorescence by distinguishing between A and G opposite the BDF base, respectively (Figure 14) [71,72]. Other pyrene-labeled pyrimidines, **^AMPy^U**, **^4′Py^T**, and **^Oxo-Py^U**, can also be used to efficiently discriminate A in a target DNA opposite the BDF base by displaying enhanced emission. Such clear fluorescence changes are very useful for SNP genotyping [73,74,75]. However, pyrene-labeled 7-deaza-2′-deoxyadenosine **^Py^A** exhibited increased emission when the bases opposite **^Py^A** were mismatched bases, and fluorescence intensity was completely quenched when a matched T was encountered [76].

## 6. Microenvironment-Sensitive Probes

Fluorescent nucleosides have been employed as microenvironment-sensitive probes, utilizing their high sensitivity to changes in polarity, viscosity, and surrounding pH. DNA or RNA containing this nucleoside has been studied for the selective detection of target DNA/RNA, abasic sites, and so on.

### 6.1. Probes Containing a Heterocycle-Conjugated Pyrimidine

**Figure 15:**
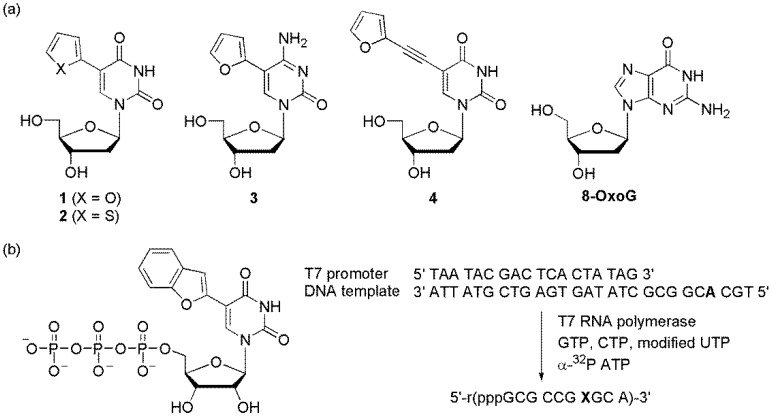
(**a**) Furan- or thiophene-modified pyrimidines and (**b**) incorporation of ribonucleoside triphosphate.

The Tor group designed furan-modified dU (**1**), thiophene-modified dU (**2**), furan-modified dC (**3**), and an extended ethynylfuran dU (**4**) as viscosity-sensitive fluorescent molecular probes, also referred to as molecular rotors (Figure 15a) [77,78]. Viscous media impeded the free rotation of these heterocycles to provide a structurally robust environment. This resulted in an increase in fluorescence intensity while reducing the contribution of the nonradiative decay pathway. Particularly, when the ODN containing furan-modified dU (**1**) was hybridized and the abasic site was located on the opposite side of **1**, a significant emission enhancement was observed compared to that of the matched duplex. Although the exact structure around the abasic site is not clear, it is believed that the intrahelical vacant but confined space between the neighboring base pairs effectively limited the free rotation of the furan-uracil single bond, resulting in an increase in fluorescence. The thiophene-modified dU (**2**) was demonstrated to be an efficient probe for the detection of G, 8-oxoG, and its transverse mutation product T by providing significantly different emission intensities [79].

The Srivatsan group synthesized benzofuran-labeled uridine and incorporated it into oligoribonucleotides using T7 RNA polymerase to produce fluorescent oligoribonucleotide constructs (Figure 15b) [80]. Abasic site-containing duplexes were constructed by hybridizing an RNA transcript to custom DNA and RNA oligonucleotides that contained an abasic-site surrogate. The abasic site-containing duplex (RNA/DNA) showed nearly a 4-fold higher emission than that of the perfect duplex. However, an RNA/RNA duplex that possessed an abasic site opposite the modified uracil showed slightly increased emission compared to that of the matched RNA-RNA duplex. In addition, they designed benzofuran-labeled 2′-deoxyuridine and demonstrated that it can discriminate an abasic site in a model depurinated sarcin/ricin RNA motif of a eukaryotic 28S rRNA [81].

### 6.2. ESF Probes

**Figure 16:**
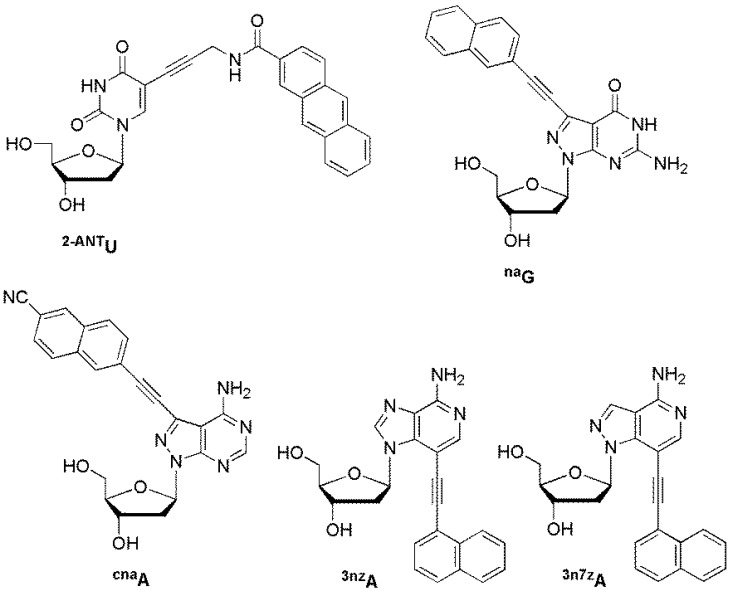
ESF probes.

The Saito group developed DNA probes containing an environmentally sensitive fluorescent (ESF) nucleoside. These probes could sense a target DNA through significant changes in fluorescence wavelength and intensity. A strong emission band appeared in polar environments. Both the emission intensity and emission wavelength of 2-anthracenecarboxamide-labeled 2′-deoxyuridine **^2-ANT^U** were significantly affected by solvent polarity (Figure 16) [82]. When a probe containing **^2-ANT^U** was hybridized with DNA targets, strong fluorescence was observed only for its perfectly matched target. C7-naphthylethynylated 8-aza-7-deaza-2′-deoxyguanosine **^na^G** exhibited very weak fluorescence and emitted at a longer wavelength of 418 nm in polar solvents such as methanol [83]. Interestingly, the emission maximum was red-shifted by 29 nm to 409 nm compared to that of other mismatched targets only when **^na^G** hybridized with a matched target DNA. The fluorescence changes caused by the differences in the opposite bases of target DNAs can be attributed to changes in the local environment around the ESF bases **^2-ANT^U** and **^na^G**.

**^cna^A**, **^3nz^A**, and **^3n7z^A** displayed significant environmentally sensitive fluorescence properties, which originate from the coplanar and non-coplanar conformers of the dye moiety and nucleobase. ODN probes containing **^cna^A**, **^3nz^A**, and **^3n7z^A** were used to sense a matched sequence and an abasic site in the target DNA through significant changes in emission wavelength and intensity [84,85,86].

#### 6.3. pH-Sensitive Probes

**Figure 17:**
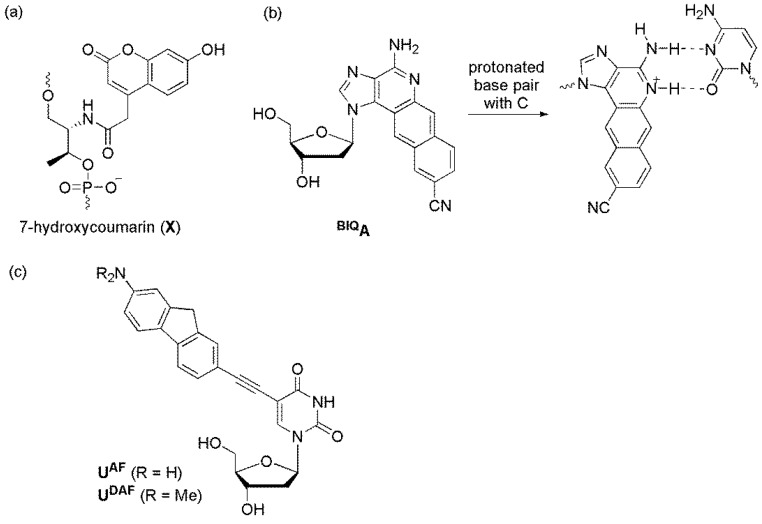
pH-sensitive probes. (**a**) 7-hydroxycoumarin; (**b**) **^BIQ^A**; and (**c**) **U^AF^** and **U^DAF^**.

The Asanuma group designed a quencher-free MB by introducing 7-hydroxycoumarin (**X**) into the stem region (Figure 17a) [87]. **X** showed quenched fluorescence upon protonation. The p*K*_a_ of **X** in a single strand was 8.8. However, it exceeded 10 in the DNA duplex because of the anionic and hydrophobic microenvironment inside the duplex. Without the target at pH 8, the stem region formed double strands, and the fluorescence was quenched. However, when the target was added, the MB opened, and the dye was deprotonated, resulting in the fluorescence of the formed duplex being 10-fold higher than that observed for MB itself.

The Saito group reported **^BIQ^A**, which exhibited large changes in absorption and fluorescence spectra upon the protonation/deprotonation of the N5 positions of **^BIQ^A**. SNP identification of a C/T (mutant/wild type) sequence of the breast cancer type 1 gene was examined using a DNA probe containing **^BIQ^A** at pH 7.5 (Figure 17b) [88]. When the opposite base of **^BIQ^A** was thymine, an intense emission band was observed. In contrast, when the opposite base was cytosine, fluorescence was significantly reduced as protonation occurred.

We synthesized 2′-deoxyuridine derivatives **U^AF^** and **U^DAF^** labeled with 2-aminofluorene and 2-dimethylaminofluorene, respectively (Figure 17c) [89]. The p*K*_a_ values of **U^AF^** and **U^DAF^** were 4.27 and 4.66, respectively, and their fluorescence increased under acidic conditions. Thus, ODNs containing **U^AF^** and **U^DAF^** exhibited distinct pH-sensitive emission behaviors upon hybridization with matched and mismatched targets [62]. ODNs bearing **U^AF^** and **U^DAF^** as a fluorescent nucleotide and C- or T-FBs, especially ODN4 bearing **U^DAF^** and C-FBs, clearly discriminated their A-matched targets, with increased emissions, under slightly acidic conditions (pH 6.5 and 6.0). However, ODN4 bearing **U^FL^** and C-FBs allowed for efficient SNP typing, regardless of the tested pH value (pH 5.5–8.0).

Table 2 summarizes the characteristics and the fluorophore used for the single-labeled probes discussed above.

## 7. Conclusions

Recently, single nucleotide polymorphism (SNP) detection techniques have evolved to become automated, efficient, and relatively inexpensive. In particular, DNA probes labeled with a single fluorophore are attractive because they are simple and inexpensive to fabricate. The following systems have been developed for this purpose: (1) A single fluorophore is connected to the ODN, and a change in fluorescence following duplex formation with the target nucleic acid is caused by the quenching effect of the guanine base adjacent to the fluorophore; (2) Cyanines, such as thiazole orange and oxazole yellow, have a property in which their fluorescence increases when intercalated into a nucleic acid duplex. This property of a cyanine dye can be used to selectively recognize matched and mismatched targets; (3) Fluorescent nucleobase analogs with structures similar to those of native nucleobases and capable of Watson-Crick base pairing have been developed and used for SNP typing; (4) Probes bearing a fluorescently labeled base exhibit dramatic emission changes after detecting a specific sequence; (5) Fluorescent nucleosides that are sensitive to polarity, viscosity, and surrounding pH have been designed and applied to SNP typing. All of these single-labeled nucleosides can be inserted at any position of the DNA according to the sequence to be designed. In addition, the biomolecules or nanoparticles can be attached to the 5′- or 3′-end of these oligonucleotide probes, increasing their applicability. Finally, no quencher is required and only a single fluorophore is included, making it possible to build simple and inexpensive probe systems. Therefore, this type of probe system has the potential to become a powerful tool for the detection of single nucleotide variations.

## Figures and Tables

**Table 1:** Quencher-free linear beacons.

ODN ^1^	Sequence ^2^	ODN ^1^	Sequence ^2^
ODN1(U^F^)	5′-d(TGGACTAU^F^ATCAATG)-3′	ODN1′(N)	3′-d(ACCTGATNTAGTTAC)-3′
ODN2(U^F^)	5′-d(TGGACTTU^F^TTCAATG)-3′	ODN2′(N)	3′-d(ACCTGAANAAGTTAC)-3′
ODN3(U^F^)	5′-d(TGGACTGU^F^GTCAATG)-3′	ODN3′(N)	3′-d(ACCTGACNCAGTTAC)-3′
ODN4(U^F^)	5′-d(TGGACTCU^F^CTCAATG)-3′	ODN4′(N)	3′-d(ACCTGAGNGAGTTAC)-3′

^1^ **U^F^** is **U^FL^**, **U^FO^**, **U^DBF^**, or **U^DBT^**. ^2^ Underlined bases are the FBs of the **U^F^** units.

**Table 2:** Single-labeled oligonucleotide probes.

No.	Probes	Notable Features	Fluorophores Used	Ref.
1	Guanine-quenching probes	Utilization the quenching effect of adjacent guanosine.	Fluorescein, BODIPY, 6-carboxyfluorescein, and tetramethyl-6-carboxyrhodamine	[15,16,17,18]
2	Cyanine-containing probes	Interaction of cyanine derivatives with nucleobases leads to the enhancement of fluorescence	Thiazole orange (**TO**), oxazole yellow (**YO**), thiazolopyridine (**MO**), and oxazolopyridine (**JO**)	[19,20,21,22,23,24,25]
3	Probes containing a fluorescent nucleobase analog	Utilization of fluorescent base analogs that are structurally similar to native nucleobases, capable of pairing with Watson-Crick pairs, and applicable as SNP probes	Flavin (**Fl**), deazaflavin (**dFl**), 3-methyl isoxanthopterin (**3-MI**), etc.	[27,28,29,30,31,32,33,34,35,36,37,38,39]
4	HyBeacon	HyBeacon probes can be integrated into real-time PCR analysis to detect specific DNA targets	6-Carboxyfluorescein, tetrachloro-6-carboxyfluorescein, and hexachloro-6-carboxyfluorescein	[43]
5	Nucleobase-labeled fluorophore with an acetylene group	Probes for SNP detection, trinucleotide repeats, etc. have been developed	Pyrene and fluorene derivatives	[44,45,46,47,48,49,50,51,52,53,54,55,56,57,58,59,60,61,62,63,64]
6	BDF probes	Clearly distinguish the type of base on the opposite strand of the BDF base	Pyrene	[71,72,73,74,75,76]
7	Probes containing a heterocycle-conjugated pyrimidine	Efficient probes for an abasic site, 8-oxoG, etc.	Furan-, thiophene-, or benzofuran-modified pyrimidines	[77,78,79,80,81]
8	ESF probes	DNA probes containing an environmentally sensitive fluorescent nucleoside	Pyrene and naphthalene derivatives	[82,83,84,85,86]
9	pH-sensitive probes	DNA probes exhibit pH-sensitive emission behaviors	7-hydroxycoumarin, benzo[*g*]imidazo[4,5-*c*]quinoline (**^BIQ^A**), and 2′-deoxyuridine labeled with 2-dimethylaminofluorene (**U^DAF^**)	[62,87,88,89]

## Supplementary Materials

Supplementary File 1
