# Supplementary figures and images for: Single-Labeled Oligonucleotides Showing Fluorescence Changes upon Hybridization with Target Nucleic Acids
Source: Molecules. 2018 Jan 8;23(1):124. doi: 10.3390/molecules23010124 (PMC6017082; doi:10.3390/molecules23010124)

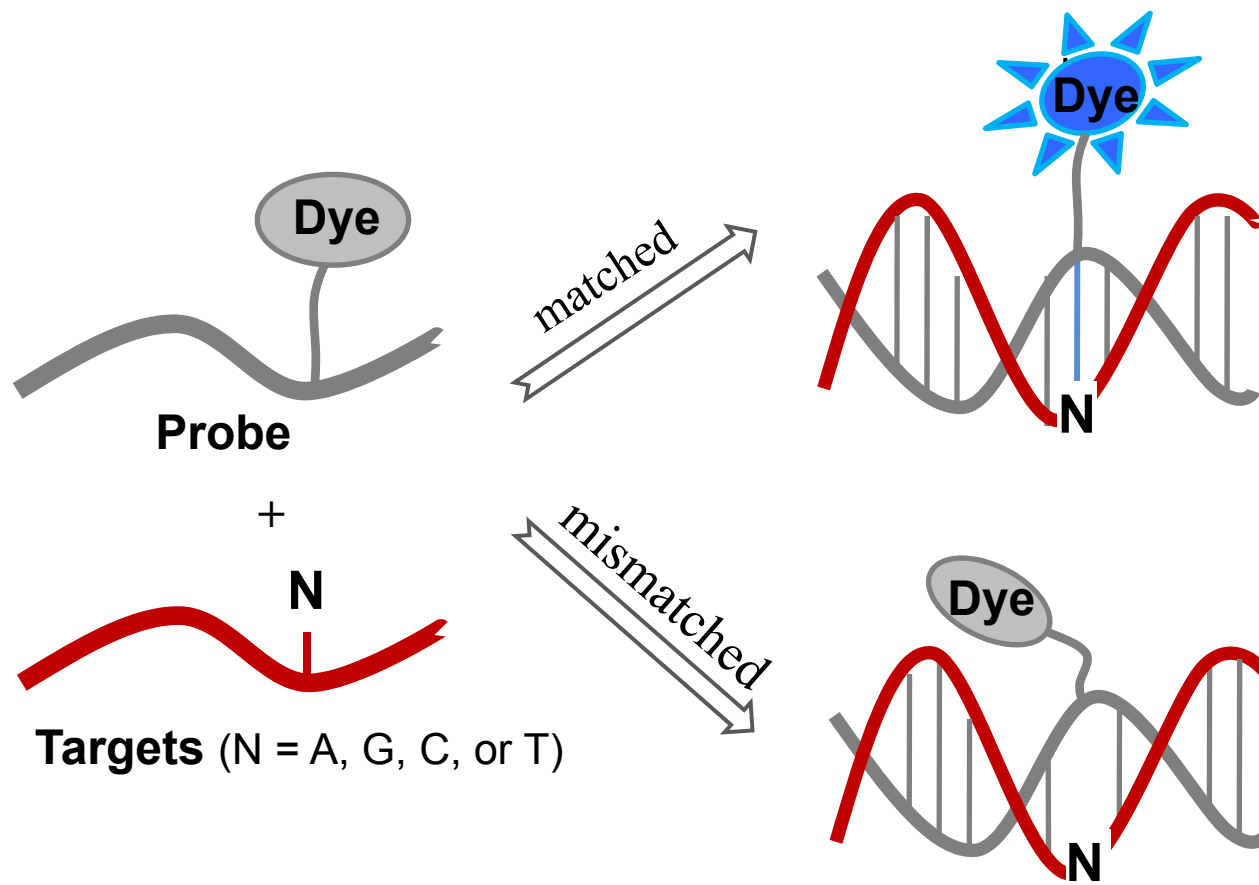

Supplement: Supplementary File 1 [file molecules-23-00124-s001.pdf]
